# Risk factors for myopic choroidal neovascularization-related macular atrophy after anti-VEGF treatment

**DOI:** 10.1371/journal.pone.0273613

**Published:** 2022-09-22

**Authors:** Ki Woong Bae, Dong Ik Kim, Bo Hee Kim, Baek-Lok Oh, Eun Kyoung Lee, Chang Ki Yoon, Un Chul Park

**Affiliations:** 1 Department of Ophthalmology, Hangil Eye Hospital, Incheon, Korea; 2 Department of Ophthalmology, Dongtan Sacred Heart Hospital, Hallym University Medical Center, Hwaseong, Korea; 3 Department of Ophthalmology, Seoul National University College of Medicine, Seoul, Korea; University of Florida, UNITED STATES

## Abstract

**Purpose:**

The study aimed to evaluate risk factors for macular atrophy (MA) associated with myopic choroidal neovascularization (mCNV) during long-term follow-up after intravitreal anti-vascular endothelial growth factor (VEGF) treatment in highly myopic eyes.

**Methods:**

The medical records of patients who received intravitreal injection of anti-VEGF agents as mCNV treatment and were followed-up for more than 36 months were retrospectively reviewed. The risk factors for the development of mCNV-MA, which is the fovea-involving patchy atrophy lesion adjacent to mCNV, were investigated using the Cox proportional hazard model.

**Results:**

A total of 82 eyes (74 patients) were included in the study. The mean age at anti-VEGF treatment was 56.3 ± 12.5 years (range, 26–77), and the mean follow-up period was 76.3 ± 33.5 months (range, 36–154). During follow-up, mCNV-MA developed in 27 eyes (32.9%), and its occurrence was estimated to be 24.5% at 3 years and 37.3% at 5 years after the first anti-VEGF treatment. Old age (hazard ratio [HR] = 1.054, 95% confidence interval [CI]: 1.018–1.091; *P* = 0.003) and greater CNV size at baseline (HR = 2.396, CI: 1.043–5.504; *P* = 0.040) were significant factors for mCNV-MA development. Eyes with a thinner subfoveal choroid were more likely to show faster enlargement of the mCNV-MA during follow-up.

**Conclusions:**

In mCNV eyes treated with intravitreal anti-VEGF agents, older age and greater mCNV size at baseline were risk factors for the development of MA during long-term follow-up, which was associated with a poor visual prognosis.

## Introduction

Pathologic myopia (PM) is a major cause of irreversible vision impairment worldwide, especially in East Asia, where myopia is highly prevalent [1–4]. One of the most severe vision-threatening complications of PM is myopic choroidal neovascularization (mCNV) [5], which is reported in 5%–11% of individuals with PM [6]. In the active stage of mCNV, the fibrovascular membrane forms around the lesion, and patients experience a sudden decrease in central vision. The mCNV scar regresses over time and leaves an area of chorioretinal atrophy, which characterizes the atrophic stage. The chorioretinal atrophic lesion enlarges over time, eventually resulting in mCNV-related macular atrophy (mCNV-MA).

Intravitreal anti-vascular endothelial growth factor (VEGF) therapy has been established as the first-line treatment for mCNV based on results of randomized clinical trials [7–9] that reported significantly improved visual prognosis compared to photodynamic therapy or sham control. However, some mCNV patients still experience central vision loss even after intravitreal anti-VEGF therapy because of mCNV-MA formation after long-term follow-up [2, 10, 11]. The area of mCNV-MA, which is characterized by the lack of Bruch membrane as observed with swept-source optical coherence tomography (OCT), also lacks photoreceptors, retinal pigment epithelium (RPE), and choriocapillaris, causing absolute scotoma [12]. However, little is known about the factors associated with mCNV-MA development after anti-VEGF therapy. In this study, we investigated the clinical features and risk factors of mCNV-MA during long-term follow-up in patients who underwent intravitreal anti-VEGF injections for the treatment of mCNV.

## Materials and methods

### Patients

This study was approved by the Institutional Review Board of Seoul National University Hospital (IRB no. 2003-231-1115) and adhered to the tenets of the Declaration of Helsinki. Informed consent was waived by IRB because of the retrospective nature of this study, and the analysis used anonymous clinical data. The medical records of highly myopic patients who underwent one or more intravitreal injections of anti-VEGF for the treatment of mCNV at the Retina Center of the Seoul National University Hospital between January 2010 and December 2018 and were followed-up for ≥ 36 months were retrospectively reviewed. High myopia was defined as eyes with axial length > 26.0 mm and/or refractive errors < −6.0 diopters in spherical equivalent. The exclusion criteria were as follows: 1) a history of any treatment for mCNV including anti-VEGF, laser photocoagulation, and photodynamic therapy; 2) presence of patchy chorioretinal atrophy adjacent to mCNV at the time of the first anti-VEGF treatment; 3) presence of glaucoma or other retinal disorders such as severe diabetic retinopathy, retinal vascular diseases, uveitis, retinal detachment, and age-related macular degeneration; and 4) a history of intraocular surgery except cataract extraction.

### Examinations

All patients underwent a complete ophthalmic examination, including best-corrected visual acuity (BCVA) assessment, refractive error, intraocular pressure measurement, slit lamp examination, and dilated fundus examination. Ocular laboratory examinations included axial length measured by IOL Master 700 (Carl Zeiss Meditec Inc., Jena, Germany), color fundus photography, ultra-widefield retinal imaging (Optos 200Tx; Optos PLC, Dunfermline, UK), and spectral-domain OCT using the Cirrus HD-OCT (Carl Zeiss Meditec, Dublin, CA). Fluorescein angiography (FA) was performed using a fundus camera (TRC-50DX; Topcon, Tokyo, Japan), and indocyanine green angiography and fundus autofluorescence images were obtained using a confocal scanning laser ophthalmoscope (HRA-2; Heidelberg Engineering, Heidelberg, Germany).

Based on color fundus photography and/or FA images, the location of mCNV was considered subfoveal when the CNV was located under the center of the fovea, juxtafoveal when the edge of CNV was located within 200 μm from the foveal center, and extrafoveal when the distance between the CNV edge and foveal center was more than 200 μm. Spectral-domain OCT scan images were also used to assess the location of the mCNV. According to the Meta-analysis of Pathologic Myopia (META-PM) Study Group classification, myopic maculopathy was classified as follows: category 1, tessellated fundus; category 2, diffuse chorioretinal atrophy; category 3, patchy chorioretinal atrophy; and category 4, macular atrophy [13]. The presence of a lacquer crack and posterior staphyloma was also recorded. Based on the ultra-widefield retinal images, the presence and type of posterior staphyloma were determined according to the Ohno-Matsui’s classification system: wide macular, narrow macular, peripapillary, nasal, inferior, and others [14]. The central foveal thickness (CFT) was obtained from the central 1-mm subfield in the macular thickness map. Subfoveal choroidal thickness (SCT), the perpendicular distance from the Bruch membrane to the sclera-choroidal junction at the subfovea, was measured manually using a caliper provided by the OCT machine. The area of mCNV at baseline was manually measured using the digitalized FA images obtained in the early phase. A dome-shaped macula was defined as an inward bulge of the macular RPE > 50 μm above a presumed line of the RPE line at the bottom of the macular curvature [15]. The presence of myopic macular retinoschisis and its progression during follow-up was determined based on B-scan OCT images [16], and macular retinoschisis was considered to have progressed when its height or extent increased during follow-up [17].

During follow-up, the development of mCNV-MA was considered when a patchy atrophy (PA) lesion, which is a gray-whitish well-demarcated round or oval lesion of chorioretinal atrophy, developed adjacent to or surrounding the mCNV and involved the fovea. Fundus autofluorescence images were also used to confirm the presence of mCNV-MA. A PA lesion not adjacent to mCNV was not considered to be related to the mCNV and was not regarded as mCNV-MA. The area of mCNV-MA was measured using color fundus photographs during follow-up. Subretinal fibrosis during follow-up was defined as a whitish or grayish fibrotic mass at the macula on fundus photography, which was observed as a well-demarcated homogenous hyperreflective lesion located between the neurosensory retina and RPE on OCT images [18].

### Treatment

Patients were treated with an intravitreal injection of anti-VEGF agents, including 1.25 mg bevacizumab (Avastin; Genentech Inc., San Francisco, CA), 0.5 mg ranibizumab (Lucentis; Novartis Pharma AG, Basel, Switzerland), or 2.0 mg aflibercept (Eylea, Bayer HealthCare, Leverkusen, Germany). When diagnosed with mCNV, the initial treatment of anti-VEGF was performed once at baseline, and additional treatments were administered on an as-needed basis. The criteria for retreatment were as follows: 1) persistence or recurrence of intra- and/or subretinal fluid on OCT, 2) new subretinal hemorrhage from the mCNV lesion, and 3) persistence or recurrence of leakage in FA. Patients were examined monthly after treatment, and the visit interval was adjusted according to disease activity, but no longer than three months.

### Data analyses

Two independent retinal specialists (DIK and BHK) blinded to the clinical information performed manual measurements of the area and thickness parameters. Any discrepancies were adjudicated by a senior retinal specialist (UCP), and the measurements from the two graders were averaged for statistical analysis. Demographic factors and ocular characteristics were compared between eyes that developed mCNV-MA during follow-up and those that did not, and a Cox proportional hazard model was used to identify the risk factors and to calculate hazard ratios for the development of mCNV-MA. Statistical analysis was conducted using the Statistical Package for the Social Sciences for Windows software (version 25.0; IBM, Armonk, NY, USA). Statistical significance was set at *P* < 0.05.

## Results

A total of 82 eyes (74 patients) were included in the study. The mean age at the time of mCNV diagnosis was 56.3 ± 12.5 years (range, 26–77). The mean axial length was 29.9 ± 2.1 mm (range, 26.8–34.4 mm), and the mean BCVA at baseline was 0.91 ± 0.41 logMAR (range, 0.00–2.00; counting fingers-20/20). The location of mCNV was subfoveal in 65 eyes (79.3%), juxtafoveal in 15 eyes (18.3%), and extrafoveal in 2 eyes (2.4%). According to the META-PM Study Group classification, at the time of mCNV diagnosis, 14 (17.1%), 51 (62.2%), and 17 (20.7%) eyes were graded as having myopic maculopathy in categories 1, 2, and 3, respectively. At baseline, we observed posterior staphyloma in 72 eyes (87.8%), lacquer cracks in 65 eyes (79.3%), and myopic macular retinoschisis in 15 eyes (18.3%).

**Fig 1:**
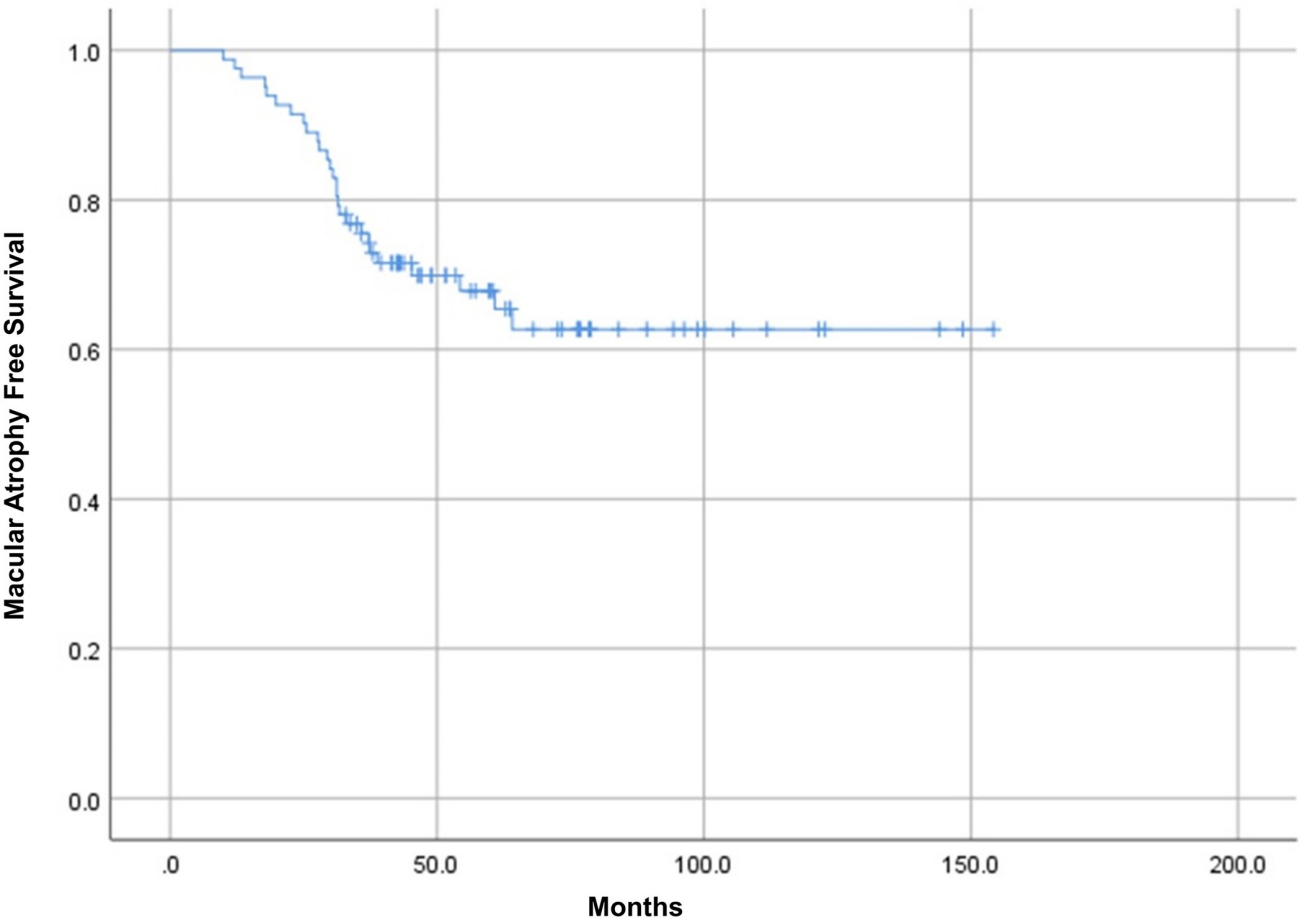
Kaplan–Meier survival curve for the development of myopic choroidal neovascularization related macular atrophy.

The mean follow-up period after the first anti-VEGF treatment was 76.3 ± 33.5 months (range, 36–154). During follow-up, mCNV recurrence was observed in 32 eyes (39.0%). The mean number of anti-VEGF injections was 4.4 ± 3.0 (range, 1–15). The development of mCNV-MA during follow-up was observed in 27 eyes (32.9%), and the mean time duration from the first anti-VEGF treatment was 31.2 ± 13.4 months (range, 10–64). Kaplan–Meier analysis estimated that the cumulative probabilities of mCNV-MA were 1.2% at 1 year, 8.5% at 2 years, 24.5% at 3 years, 30.1% at 4 years, and 37.3% at 5 years after the first anti-VEGF treatment (Fig 1).

**Fig 2:**
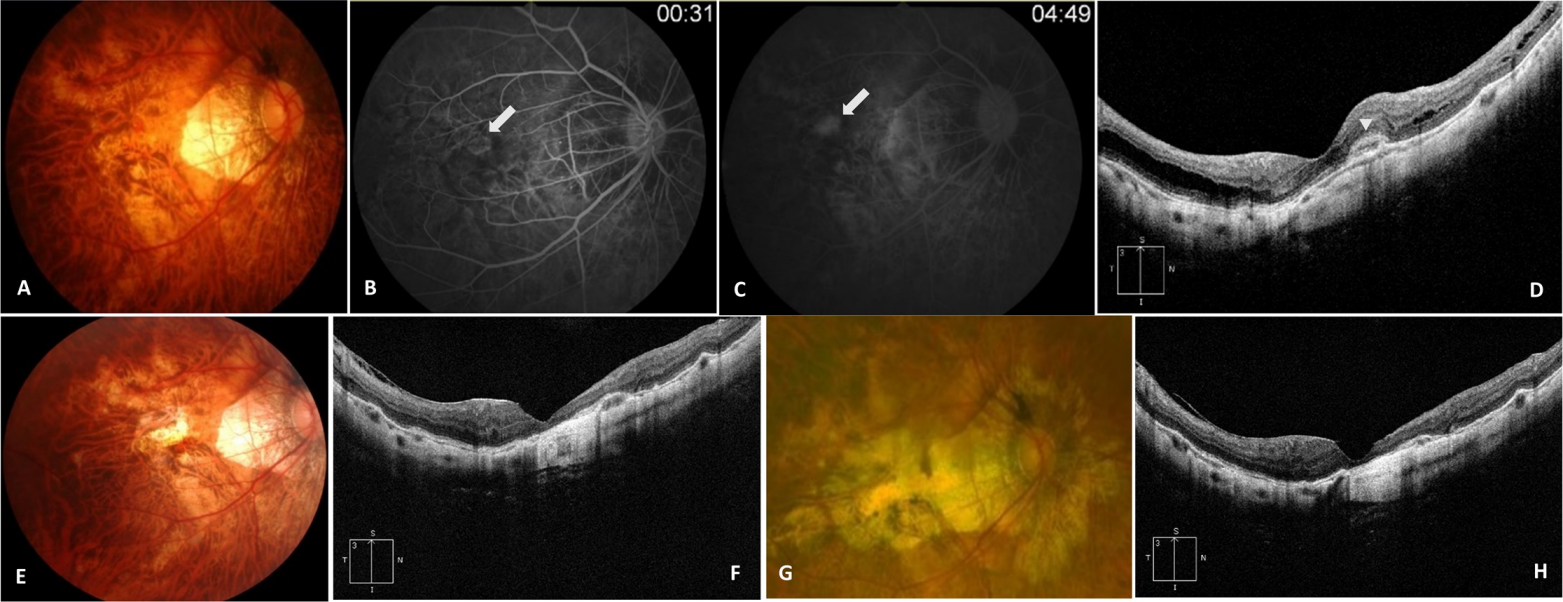
Representative images of a 62-year-old woman who developed myopic choroidal neovascularization (mCNV) related macular atrophy (MA) after anti-vascular endothelial growth factor (VEGF) treatment. **(A)** Color fundus photograph showed subretinal hemorrhage of the right eye. The best-corrected visual acuity (BCVA) was 20/160 at baseline. (**B, C)** Early phase fluorescein angiogram (FA) image showed hyperfluorescence at the juxtafoveal mCNV, and leakage from the mCNV was observed in late phase image of FA (white arrows). (**D)** Spectral domain optical coherence tomography (OCT) images showed hyperreflective lesion corresponding to mCNV (white arrowhead). **(E)** At 28 months after the first anti-VEGF treatment, mCNV-MA was observed as a whitish well-demarcated chorioretinal atrophy adjacent to the mCNV. She underwent two more injections of anti-VEGF during the period. **(F)** Loss of retinal pigment epithelium (RPE) and hyperreflective line of ellipsoid zone was observed at the fovea in the OCT image. The size of mCNV-MA was 0.51 mm^2^. (**G)** At the final follow-up, which was 92 months after the initial observation of the mCNV-MA, the area of mCNV-MA increased to 2.74 mm^2^. During the period, she underwent four more injections of anti-VEGF. The final BCVA was 20/200. **(H)** In the OCT image, loss of outer retinal structure and RPE was more pronounced compared to **(F)**, and subretinal fibrosis was observed as well-demarcated hyperreflective mass.

**Fig 3:**
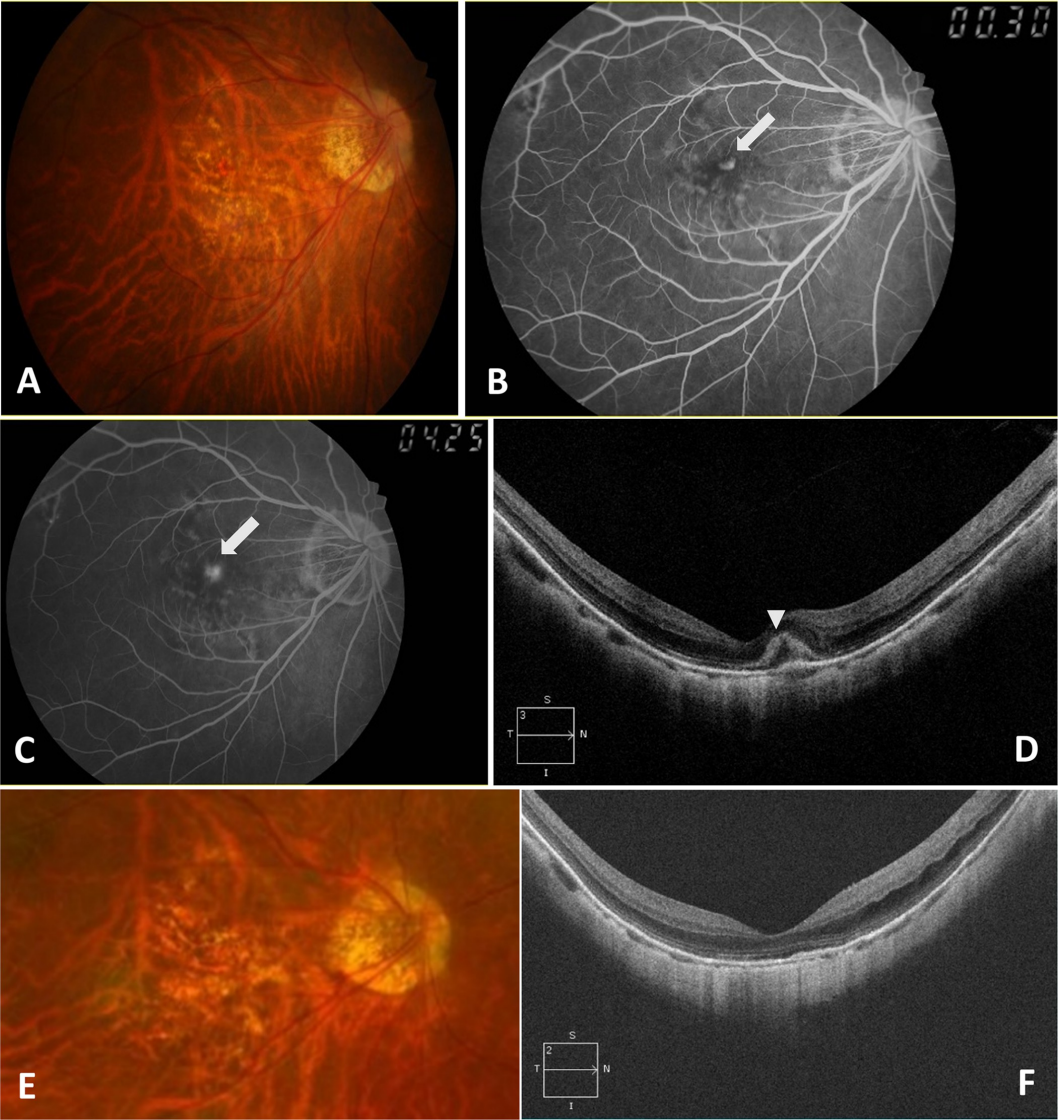
Representative images of a 57-year-old woman who did not develop myopic choroidal neovascularization (mCNV) related macular atrophy after anti-vascular endothelial growth factor (VEGF) treatment. **(A)** Color fundus photograph showed subretinal hemorrhage of the right eye. The best-corrected visual acuity (BCVA) was 20/80 at baseline. (**B, C)** The mCNV was observed as a hyperfluorescent lesion located at the subfovea in the early phase image of fluorescein angiography (FA), and leakage from mCNV was observed in late phase FA image (white arrows). (**D)** A spectral domain optical coherence tomography (OCT) showed hyperreflectivity corresponding to subretinal hemorrhage and mCNV (white arrowhead). (**E)** At the final follow-up visit, which was 78 months after the first anti-VEGF treatment, increased lacquer cracks were observed, and BCVA was 20/63. **(F)** No mCNV and subretinal fibrosis were observed in the OCT image.

The demographics and clinical characteristics of the patients according to the development of mCNV-MA are presented in Table 1. Patients who developed mCNV-MA during follow-up were older at baseline (*P* = 0.001), had a longer follow-up period (*P* = 0.002), and had more anti-VEGF treatments during follow-up (*P* = 0.034) compared to those who did not develop mCNV-MA (Table 1). However, there were no significant differences in refractive errors, axial length, BCVA, CFT, SCT, CNV location, myopic maculopathy grades, and the prevalence of posterior staphyloma, lacquer crack, dome-shaped macula, and MTM at baseline, and CNV recurrence during follow-up. During follow-up, the mean BCVA changed from 0.89 ± 0.41 to 1.02 ± 0.65 logMAR (*P* = 0.243) in eyes that developed mCNV-MA and from 0.74 ± 0.54 to 0.66 ± 0.53 logMAR (*P* = 0.172) in eyes that did not develop mCNV-MA. The mean final BCVA of eyes that developed mCNV-MA was significantly worse than that of eyes that did not (*P* = 0.010). Progression of myopic macular retinoschisis was observed in 4 of 27 eyes (14.8%) that developed mCNV-MA during follow-up and 11 of 55 eyes (20.0%) that did not (*P* = 0.763). Representative cases with and without mCNV-MA development during follow-up are shown in Figs 2 and 3, respectively.

**Table 1:** Demographics and clinical characteristics of eyes with and without myopic choroidal neovascularization related macular atrophy.

Factors	Total (n = 82)	MA Group (n = 27)	Non-MA Group (n = 55)	*P* value
Age, years	56.3 ± 12.5	63.0 ± 8.3	53.0 ± 12.9	0.001
Sex, female (%)	70 (85.4)	23 (85.2)	47 (85.5)	1.000
Follow-up period, months	76.3 ± 33.5	92.9 ± 33.4	68.2 ± 30.6	0.002
Refractive errors in SE, D	-13.9 ± 6.4	-13.0 ± 5.5	-14.3 ± 6.9	0.739
Axial length, mm	29.9 ± 2.1	30.0 ± 1.5	29.7 ± 2.6	0.723
Number of anti-VEGF injection	4.4 ± 3.0	5.0 ± 2.4	4.1 ± 3.2	0.034
Baseline BCVA, logMAR	0.91 ± 0.41	0.89 ± 0.41	0.74 ± 0.54	0.067
Baseline CFT, μm	301.3 ± 64.0	290.3 ± 55.4	306.8 ± 67.7	0.425
Baseline SCT, μm	48.4 ± 38.6	43.4 ± 28.4	50.9 ± 42.8	0.632
CNV location, subfoveal/non-subfoveal (%)	65/17 (79.3/20.7)	20/7 (74.1/25.9)	45/10 (81.8/18.2)	0.563
Grade of myopic maculopathy at baseline (%)				0.470
Grade 1 (Tessellated fundus)	14 (17.1)	3 (11.1)	11 (20.0)	
Grade 2 (Diffuse atrophy)	51 (62.2)	17 (63.0)	34 (61.8)	
Grade 3 (Patchy atrophy)	17 (20.7)	7 (25.9)	10 (18.2)	
Staphyloma at baseline (%)	72 (87.8)	25 (92.6)	47 (85.5)	0.485
Type of staphyloma at baseline				0.864
Wide macular	53 (73.6)	19 (76.0)	34 (72.3)	
Narrow macular	16 (22.2)	6 (24.0)	10 (21.3)	
Peripapillary	2 (2.8)	0 (0.0)	2 (4.3)	
Inferior	1 (1.4)	0 (0.0)	1 (2.1)	
Lacquer crack at baseline (%)	65 (79.3)	24 (88.9)	41 (74.5)	0.158
Dome shaped macula at baseline (%)	19 (23.2)	5 (18.5)	14 (25.5)	0.484
Myopic macular retinoschisis at baseline (%)	15 (18.3)	8 (29.6)	7 (12.7)	0.075
CNV size at baseline, mm^2^	0.50 ± 0.46	0.62 ± 0.55	0.45 ± 0.40	0.081
CNV recurrence during follow-up (%)	32 (39.0)	13 (48.1)	19 (34.5)	0.335

MA, macular atrophy; SE, spherical equivalent; VEGF, vascular endothelial growth factor; BCVA, best corrected visual acuity; logMAR, logarithm of the minimum angle of resolution; CFT, central foveal thickness; SCT, subfoveal choroidal thickness; MTM, myopic traction maculopathy; CNV, choroidal neovascularization.

Considering various follow-up periods among patients, the Cox proportional hazard model was used to evaluate risk factors for mCNV-MA development. Older age at baseline (hazard ratio [HR] = 1.054, 95% confidence interval [CI]: 1.018–1.091; *P* = 0.003) and greater CNV size at baseline (HR = 2.396, 95% CI: 1.043–5.504; *P* = 0.040) were identified as risk factors for mCNV-MA development.

To identify the clinical factors associated with the enlargement of mCNV-MA, 25 eyes that were followed up for more than 12 months after mCNV-MA development were divided into two groups according to the enlargement rate of mCNV-MA per year: the enlargement group (n = 13), which showed an increase in the area of mCNV-MA at ≥20%/year compared to baseline, and the non-enlargement group (n = 12), which showed an increase of <20%/year. For the measurement of mCNV-MA, interclass coefficients (ICC) showed high interobserver repeatability (ICC = 0.989, 95% CI, 0.977–0.995; *P* < 0.001). Table 2 summarizes the clinical features of eyes with and without mCNV-MA enlargement during follow-up. Baseline characteristics at the time of mCNV diagnosis were comparable between groups. At the time of mCNV-MA development, the enlargement group had significantly lower SCT compared to the non-enlargement group (20.6 ± 17.1 vs 41.2 ± 22.4 μm; *P* = 0.018), while other parameters were comparable. Using linear regression analysis, association between the increase in the area of mCNV-MA and the SCT at the time of mCNV-MA was investigated for the 25 eyes with mCNV-MA, but no statistical significance was observed (*P* = 0.117).

**Table 2:** Demographics and clinical characteristics of eyes with macular atrophy.

Factors	Enlargement Group (n = 13)	Non-enlargement Group (n = 12)	*P* value*
Baseline characteristics			
Age, years	62.6 ± 8.7	62.6 ± 8.0	0.883
Sex, female (%)	11 (84.6)	10 (83.3)	1.000
Refractive errors in SE, D	-14.3 ± 5.6	-11.9 ± 5.9	0.524
Axial length, mm	30.6 ± 2.0	29.5 ± 0.9	0.105
Number of anti-VEGF injection	5.1 ± 2.6	4.7 ± 2.3	0.815
BCVA, logMAR	1.01 ± 0.52	0.79 ± 0.24	0.294
CFT, μm	305.7 ± 71.5	272.4 ± 30.0	0.140
SCT, μm	35.7 ± 26.2	54.9 ± 30.0	0.089
CNV location, subfoveal/non-subfoveal (%)	9/4 (69.2/30.8)	10/2 (83.3/16.7)	0.645
Grade of myopic maculopathy (No. of eyes with Grade 1/2/3)	2 / 9 / 2	1 / 7 / 4	0.608
Staphyloma (%)	13 (100.0)	10 (83.3)	0.220
Lacquer crack (%)	12 (92.3)	41 (74.5)	0.593
Dome shaped macula (%)	1 (7.7)	4 (33.3)	0.160
Myopic macular retinoschisis (%)	5 (38.5)	2 (16.7)	0.378
CNV size, mm^2^	0.69 ± 0.71	0.52 ± 0.30	0.905
CNV recurrence (%)	6 (46.2)	5 (41.7)	1.000
Clinical characteristics at the time of MA development			
BCVA, logMAR	0.88 ± 0.58	0.78 ± 0.54	0.657
CFT, μm	248.5 ± 64.2	263.9 ± 68.6	0.565
SCT, μm	20.6 ± 17.1	41.2 ± 22.4	0.018
Subretinal fibrosis (%)	9 (69.2)	10 (83.3)	0.645
Myopic macular retinoschisis (%)	7 (53.8)	4 (33.3)	0.428
MA size, mm^2^	0.94 ± 0.86	1.67 ± 1.81	0.347
Clinical parameters at last visit			
BCVA, logMAR	1.14 ± 0.73	0.91 ± 0.62	0.530
CFT, μm	247.5 ± 81.9	265.6 ± 64.3	0.512
SCT, μm	7.4 ± 18.2	23.4 ± 16.1	0.005
Myopic macular retinoschisis (%)	5 (38.5)	3 (25.0)	0.673
MA size, mm^2^	7.72 ± 4.95	2.60 ± 2.39	0.002

*P value was obtained from non-parametric tests: Fisher’s exact test for categorical variables and Mann-Whitney *U* test for continuous variables.

SE, spherical equivalent; VEGF, vascular endothelial growth factor; BCVA, best corrected visual acuity; logMAR, logarithm of the minimum angle of resolution; CFT, central foveal thickness; SCT, subfoveal choroidal thickness; CNV, choroidal neovascularization; MA, macular atrophy.

## Discussion

The results of this study show that the development of MA increases as the follow-up period increases in highly myopic eyes with mCNV treated with intravitreal anti-VEGF injection, and its occurrence was estimated to be 24.5% at 3 years and 37.3% at 5 years after initial treatment. The risk factors for mCNV-MA development were older age and greater size of mCNV at baseline and a thinner subfoveal choroid was associated with the faster enlargement of mCNV-MA during follow-up.

The long-term prognosis of untreated mCNV is poor, showing progressive visual loss due to the expansion of chorioretinal atrophy. In a long-term study by Yoshida et al., approximately 90% of patients had a visual acuity of ≤20/200 at 5 years after the onset of mCNV, and a gradual decrease in visual acuity during follow-up was associated with an increased incidence of chorioretinal atrophy around the regressed mCNV, which was 74.1% at 3 years after the onset of mCNV and almost 100% after 5 years or more [19]. Although the influence of intravitreal anti-VEGF treatment on the development of chorioretinal atrophy is still elusive, it seems that the application of anti-VEGF treatment has decreased the incidence of chorioretinal atrophy associated with mCNV compared to untreated natural course, varying from 30% to 73% in recent studies [20–23]. The cumulative probabilities of mCNV-MA of 24.5% at 3 years and 37.3% at 5 years after initiation of anti-VEGF treatment in this study are also similar to the results of recent studies on chorioretinal atrophy after mCNV treated with anti-VEGF.

Even in eyes with mCNV successfully treated with intravitreal anti-VEGF injection, the initial visual gain achieved tends to decline during the follow-up [5, 24], and the development of chorioretinal atrophy related to mCNV and its macular involvement is associated with central vision loss [20, 25]. Visual outcome in eyes that developed mCNV-MA during follow-up was significantly worse than those that did not in this study. In previous studies on chorioretinal atrophy after mCNV treatment, criteria for “chorioretinal atrophy” varied, and some included both diffuse and patchy atrophy, while others included only patchy atrophy as chorioretinal atrophy [20, 22, 23]. In this study, we evaluated the incidence and risk factors of fovea-involving PA associated with mCNV after anti-VEGF treatment only. Changes in diffuse atrophy and development of PA not associated with mCNV were not considered as endpoints in the analysis because they are not associated with central vision loss or scotoma. In contrast, mCNV-MA, which is characterized by macular Bruch membrane defects, also lacks RPE, choriocapillaris, and photoreceptors in the corresponding area and is of great importance for long-term visual prognosis [12, 26].

In previous studies, old age, large CNV size, and subfoveal location of CNV were identified as risk factors for chorioretinal atrophy development after anti-VEGF treatment for mCNV [21–23, 27]. In this study, the Cox proportional hazard model identified old age and greater CNV size as risk factors for mCNV-MA. Although it remains unclear how these factors are associated with mCNV-MA development, decreased RPE function due to aging may be associated with vulnerability to MA development [27]. In eyes with a larger size of mCNV, its shrinkage may result in centripetal contraction of the RPE over a greater area adjacent to mCNV compared to eyes with a smaller size [22, 27]. In addition, large mCNV is more likely to have a greater area of edema and hemorrhage around the CNV, and the retinal toxicity from these factors might be associated with a higher risk of mCNV-MA during follow-up [27, 28]. However, subfoveal location of mCNV was not identified as a risk factor for mCNV-MA in this study. Although the reason for this discrepancy is unclear, it seems to be associated with a much longer mean follow-up period of 76.3 months in this study compared to previous studies. Due to progressive enlargement of chorioretinal atrophy once developed, PA lesions secondary to non-subfoveal mCNV would also involve the macula when followed-up for a long period.

In this study, mCNV-MA enlargement during follow-up was associated with a thin subfoveal choroid at the time of mCNV-MA. In highly myopic eyes, choroidal thinning has been reported to be closely associated with mCNV development [29, 30], and choroidal thickness decreases following anti-VEGF treatment [31]. Although criteria for enlargement were arbitrary, this result suggests that subfoveal choroidal thinning, which may represent the compromised choroidal perfusion in the subfoveal region, may also play an important role in the long-term change of mCNV-MA once it has developed. Even without mCNV, it is possible that the Bruch membrane in highly myopic eyes with an extremely thin choroid is fragile and prone to develop defects [32]. However, a tendency toward a significant difference was observed in baseline OCT between the enlargement and non-enlargement groups, and this may have partly influenced the association between the enlargement of mCNV-MA and thinner choroid at the time of mCNV-MA development. Further study with larger sample size is needed to confirm this association. The association of inferior choroidal thickness with mCNV change or chorioretinal atrophy development after anti-VEGF treatment has also been reported [23, 33], but we did not evaluate choroidal thicknesses at other macular regions in this study.

In addition to its retrospective design, this study has several limitations. First, various anti-VEGF agents, including bevacizumab, ranibizumab, and aflibercept, were used in this study. Several studies reported that visual outcomes were comparable among these agents when used for mCNV treatment [34–36]. However, their long-term influence on mCNV-MA development is unknown, and further study is warranted. Second, the time point at which mCNV-MA was developed could not be exactly specified due to its retrospective design. Third, a relatively small number of patients were included in this study, and subgroup analysis may not be able to identify clinical factors for MA enlargement.

In conclusion, the development of MA increased during long-term follow-up after anti-VEGF treatment in eyes with mCNV and was associated with poor visual outcomes. Older age and greater size of mCNV at baseline were associated with the development of mCNV-MA, and its enlargement during further follow-up was associated with a thinner subfoveal choroid. These factors could be useful in predicting the long-term prognosis of mCNV treated with intravitreal anti-VEGF injections.

## Supporting information

S1 Data (XLSX)
